# Food, flavouring and feed plant traditions in the Tyrrhenian sector of Basilicata, Italy

**DOI:** 10.1186/1746-4269-2-37

**Published:** 2006-09-07

**Authors:** Paolo Maria Guarrera, Giovanni Salerno, Giulia Caneva

**Affiliations:** 1 Museo Nazionale Arti e Tradizioni Popolari, Piazza Marconi 8/10 00144 Rome, Italy; 2 Dipartimento di Biologia, Università di Roma Tre, Viale Marconi 446, 00146 Rome, Italy

## Abstract

**Background:**

Research was carried out in the years 2002–2003 into food, flavouring and feed folk traditions of plants in the Tyrrhenian part of the Basilicata region (southern Italy). This area was colonized in ancient times by Greeks. Data was collected through field interviews, especially of farmers.

**Methods:**

Field data were collected through structured interviews. The informants, numbered 49, belonged to families which had strong links with the traditional activities of the area.

**Results:**

61 taxa are cited, belonging to 26 botanical families, amongst which 44 used as food or flavouring and 22 for animal alimentation. Besides 7 taxa are involved in rituals especially connected with agriculture and plant growth.

**Conclusion:**

The preservation of some rituals especially concerning agricultural plants is noteworthy in the area, together with a certain degree of continuity in food uses. Knowledge and rediscovery of recipes in human and animal diet could represent an economic potential for the area.

## Background

The aim of this paper is to contribute to our knowledge of the food, flavouring and feed use, both past and present, of plants found throughout the Tyrrhenian part of Basilicata, an area never before fully investigated from this aspect. Some investigation was carried out in Basilicata region, concerning food and flavouring plants, while no research was made on fodder plants. Few data were reported for the Tyrrhenian coast in a book by Caneva et al. [1]; other papers, by Pieroni et al. [2-5], studied some communities of Arbëreshë in internal areas of the region.

**Figure 1:**
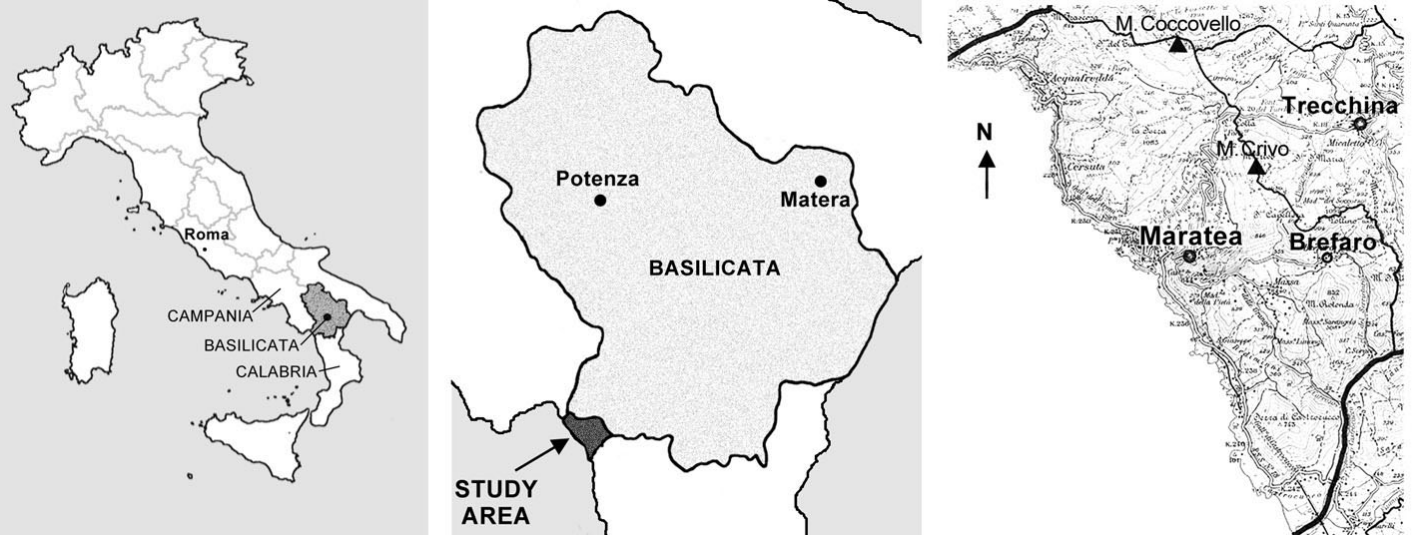
Map of the research area.

The research area is located in Potenza province, between the regions of Campania to the north and of Calabria to the south, including roughly 28 km of coastline. This territory (latitude 40°N, longitude 15°45'E) occupies an area of 105,03 km^2^, facing the Gulf of Policastro; it is rich in promontories rising straight from the sea and imposing mountains (max. 1505 m). The research was carried out in the territory of Maratea with some small scattered centres stretching from the coast to the slopes of Mt. S.Biagio (Massa, Brefaro, S. Caterina, Fiumicello) and in the area of Trécchina, in the interior (Fig. 1). The territory is covered above all with a Mediterranean maquis, a vegetation of sunny rocks and mixed woods. Old specimens of *Olea europaea, Ceratonia siliqua, Pistacia terebinthus, Quercus ilex *and *Quercus virgiliana *are found [6]. The climate is coastal Mediterranean with mild temperatures throughout the year (average yearly temperature = 14–16°C) and with a dry summer period. Rainfall is abundant (about 1200 mm per year)[6].

Total population of the studied area is 7600 inhabitants (Maratea 5000 inhabitants; Trecchina 2600)[7]. Agricultural works (production of wine, olive oil and vegetables) and pastoral activities continue, also if a good part of the economy is characterized by tourism. The Greeks were probably the first to colonise the area and to found the small town of Maratea in the VIII century B.C.

## Methods

Field data were collected during the periods April – July 2002 and March 2003 through structured interviews. The informants interviewed numbered 49 (26 men, 23 women) whose ages ranged from 25 to 97 and who mainly belonged to families which had strong links with traditional activities of the area. Most of the interviewees (36) were aged over 50, of whom: 5 were between 50 and 59, 12 between 60 and 69, 16 between 70 and 80, and 3 over 90 years old. Among the informants 19 were farmers, the others mainly building workers, restaurateurs, shepherds and housewives. Interviews were carried out using fresh plant specimens, or by going with the informants to collect plants related to folk uses. Voucher specimens of the plants were collected and deposited in the Herbarium of the University of Roma Tre. Some tape-recordings are also kept in the National Museum of Arts and Folk Traditions of Rome. The informants were requested to furnish for each plant: vernacular name, folk use (in human/animal nourishment and in rituals), the preparation and parts used, period of gathering, related recipes, and an indication of whether these cited uses were still practised. They were also asked to indicate whether the use was "personal" and/or familiar (that is practised by the informant or by one or more members of the same family) and/or practised by others (e.g. friends, acquaintances). Further data consisted of an indication, in the informant's opinion, of the frequency or rarity of a given use, even if no longer practised. The nomenclature of the listed plants follow Pignatti [8].

## Results and discussion

The results of our study are reported in Table 1 ' [see Additional file 1]'. Plant families are listed in systematic order; within each family the species are cited in alphabetical order. The taxa cited are 61, belonging to 26 botanical families, among which 44 as food or flavouring, 22 in animal alimentation and 7 in rituals especially connected with agriculture and plant growth. Number of citations and relevant percentage on total number of citations (336) were reported too. In our paper we have compared the data collected with data present in ethno-botanical texts (see references). This has been done so as to highlight uses not previously reported or possibly described for other Italian areas. Comparison with the main papers on Basilicata [1-3] that were not concerning feed plants shows that 19 plants related to ethno-botanical uses (*) are new for the region, while 23 plants (°)(Table I) ' [see Additional file 1]' are cited in the texts [1-3] but here have at least one new or different use. The food or aromatic plants found number 44 in total, 34 of these being wild. The total number of plants reported represent 19.13 % of the food and flavouring species described by Caneva et al. [1] for Basilicata region, even though some plants we describe are new for Basilicata (*Asphodeline liburnica*, *Crithmum maritimum, Cynodon dactylon, Helichrysum italicum, Inula crithmoides, Pisum sativum *subsp. *elatius*, *Sonchus tenerrimus*, *Sonchus oleraceus*, *Teucrium polium *subsp. *capitatum, Urospermum picroides*). A certain number of these are cited in specific Italian books on wild plants as food [9,10].

### A) Food and flavouring uses

Table 1 ' [see Additional file 1]' shows that the food use of wild plants is again still present and that the greatest quantities of wild herbs are harvested in spring, with the resumption of rural tasks (weed killing on farms, preparation of the vineyards). Fewer food plants are gathered during the summer, while a certain number of these are available all the year round.

The consumption of so-called "misca", a mixture of vegetables, is very frequent in the area. These greens must be boiled, strained, then cooked again in a frying pan, with garlic, beans and, in some cases, pigskin. The dish is made using the leaves of many wild plants, such as *Beta vulgaris *subsp. *vulgaris*, *Borago officinalis*, *Cichorium intybus*, *Foeniculum vulgare *subsp. *piperitum*, *Papaver rhoeas*, *Picris *sp.pl., *Sonchus *sp.pl., *Reichardia picroides *etc., to which certain cultivated species may be added, such *Solanum tuberosum *tubers. This mixture of greens, typical of the area and appealing in its simplicity, is fit to be served in restaurants and local farm guesthouses.

Pharmacobotanical properties for the wild food plants are under study [2,11,12], but several food species have been insufficiently investigated. The food use of *S. oleraceus *and *Sonchus *sp. pl. is justified by the high content of vitamin C, carotenoids, fatty acids of type ω-3 [13-16]; protective properties against hepatic injury has been described for *S. arvensis *[17].

*Origanum heracleoticum*, a species with more a valuable aroma than *Origanum vulgare *[8] and used very often in cookery, is undoubtedly fit to be cultivated in Maratea.

In the last few years, in addition to the known antibiotic activity and to the antioxidant properties of some aglicones such as thymolquinone, thymol, eugenol, 2-phenylethanol, carvacrol, 3-hexen-1-ol [18], it has been proved that the essential oil acts as an inhibitor of carcinomas [19]. The biochemical composition of the species in Calabria, a few kilometres from the area of the research and the chemosistematic of the genus *Origanum *were investigated [20,21].

The use of *Helichrysum italicum *to flavour sauces is new for Italy, but some aromatic uses have been described [22,23]; scientific literature reports its medicinal properties among which that digestive [24-26]. The plant, containing a complex of flavonoids (elicrisin), an essential oil with pinene, eugenol, nerol, caffeic and ursolic acid [27], should be more greatly appreciated for its anti-inflammatory [28,29] and antibiotic [26] properties.

The custom of eating *Inula crithmoides *tops in salad is not common in Italy; this plant and *Crithmum maritimum *are perceived as being similar and are given the same phytonim "crìtini" [26,30,31]. *C. maritimum *(potassium salt, pectin, vitamins A, B2, C, essential oil with pinene, dipentene, eugenol, carvacrol) is an anabolic, carminative, choleretic, diuretic, eupeptic and vermifuge agent [32]. Also *I. crithmoides *contains terpenes (α- and β-phellandrene, p-cymene, α- and β-pinene), in addition to linalool, carvacrol [33], flavonols [34], thymol derivatives [35].

A rarer food use is that of *Lathyrus sylvestris *(tender seeds eaten in soups) [36]. Its vernacular name "òleca" refers to a plant eaten as a vegetable, from the Latin "olera", but the toxicity of some species of the genus *Lathyrus *is known. The pathology of lathyrism is due to a toxic compound, β-amino-propionitrile [31,37].

The local practice of keeping dried figs skewered and held together with flavouring sticks is also reported for the nearby Mt. Pollino [38]. The aromatizing properties of *Myrtus communis*, containing mirtenol, tannins, terpenes, bitter compound [39] have been known since ancient times: mortadella derives, in fact, from "mortarum", a Roman sausage with myrtle [1]. The salami, ancient "lucànica", seasoned with spices and laurel fruits, of which Apicio (I sec B.C.) describes a recipe, has existed since Roman times [1].

The food use of *Leopoldia comosa *bulbs, widespread in Turkey [40], Greece [41,42] and Southern Italy [1,2,4,37,38], with its prevalently Greek culture, could have been introduced by the colonisers of Magna Grecia [43]. In the area studied, the bulbs (once sold) are consumed in various ways, flavoured with garlic, chilli pepper, mint and oregano. These bulbs are cooked after eliminating the external skin [37], boiled for a long time (in water and vinegar) to reduce the bitter taste, due to homoisoflavones [44,45] and, probably, their toxicity, as the plant could contain polyhydroxylated pyrrolizidine alkaloids [46]. Also in *Clematis vitalba *buds, the irritant compound protoanemonin is broken down through boiling.

*Borago officinalis*, an emollient agent (mucilages, allantoin and mineral salts [48]), is another species with remarkable gastronomic potential, containing small amounts of pyrrolizidine alkaloids [48]. The food use in omelettes or fritters of *Asphodeline liburnica *young buds, "sbaràvule", whose vernacular name likens it to a wild asparagus, is neither described for Italian areas nor cited by Kunkel [31,36,37,47]. The custom of eating boiled shoots of *Equisetum telmateja *(not cited in [49]) is rare in Italy [1,26].

Some plant-based dishes were (and still are) obligatory on important religious occasions: on Easter Sunday and the following day the traditional lunch is roast lamb with *L. comosa *and potatoes with chopped tomatoes, onion and black pepper. A discontinued use, instead, related to the Holy Saturday, was the consumption of a soup of cooked wild chicory with salted pigs' trotters. *Asparagus acutifolius*, in addition to being consumed in omelettes or in sauces, is included in small round loafs, "pagnottedde i spàgari" [50]. Other peculiar uses were coffee prepared with toasted seeds of *Cicer arietinum *or with the toasted rhizomes of *Cynodon dactylon*. In times of famine the starch obtained grinding the latter was added to flour to integrate it, or else the rhizomes were boiled and eaten as vegetables (uses not reported by Aliotta [49]).

### B) Fodders

Many plants are administered to herbivores such as arboreal species, Graminaceae and Leguminosae species. *Ceratonia siliqua *is one of the most widely used plants for herbivores [51,52]; in the past, this was an important economic plant on the southern Mediterranean horizon. The harvesting of *Psoralea bituminosa*, a vermifuge [53] and vulnerary agent [54], is worthy of note; however, it contains photosensitizing furocoumarins [55-57]. In pig feed we find both thick oily leaves, such as *Tussilago farfara*, *Borago officinalis*, *Nasturtium officinale, Picris echioides*, as well as *Quercus *acorns, but also *Arum italicum *and *Asphodelus microcarpus *tubers, *Crataegus monogyna *and *Pyrus amygdaliformis *fruits. Perhaps the tastiness of the famous "lucaniche" can also be attributed to the ingestion of these plants. For feeding rabbit *Foeniculum vulgare *subsp.*piperitum, Papaver rhoeas*, *Digitaria sanguinalis, Reichardia picroides, Salix alba *are indicated; only *Urtica dioica *and *P. rhoeas *are bird seed [58]. Until a few decades ago, 50% of fodder for pigs came from wild plant resources (acorns, *Arum italicum *tubers, wild fruits etc.); today this practice is still present but in a more limited way.

### C) Beliefs

Among the most interesting beliefs is that concerning *Arum italicum*: its abundant flowering and fructification is interpreted as predicting a future good harvest. In fact people think that fruits, spadix, leaves and bulb represent respectively the harvests of maize, cereals, vegetables and potatoes [59]. These fertility omens are most probably to be related to humidity levels which increases the growth and the luxuriance of certain plants. A similar belief is held also relating to *Asphodelus microcarpus*. The ancient associated the arum-like motifs with snakes in a mystical chthonic concept [60]. Finally, by attributing to spikelets of *Lolium multiflorum *subsp. *gaudini *and *Lolium perenne*, the values "much", "a little" and "nothing" on a cyclical basis, people believed it was possible to tell if their love was reciprocated. It was the last spikelet that gave the definitive answer.

## Conclusion

Data shows that many food and feed uses are still practised and that various phytonims or recipes date back to ancient uses. For example, the "làgana", of which also the Latin poet Horace speaks ("porri et ciceris...laganique catinum": Satira VI, l libro, v.115) was already known during Roman times. This dish is still prepared today in Maratea and bears the name of "cìciri e làgani": the "lagana" is a homemade pasta without eggs, suitable for serving with chick peas.

In this perspective knowledge and rediscovery of recipes in human and animal diet could represent an economic potential for the area.

The preservation of some rituals especially concerning agricultural plants is also noteworthy in the area.

## Competing interests

The author(s) declare that they have no competing interests.

## Authors' contributions

The field work for data collection was carried out above all by G.Salerno. Data analysis and manuscript preparation were conducted by all authors, but particularly by P. M. Guarrera.

## Supplementary Material

Additional File 1 Food, flavouring and feed folk uses of plants in the Tyrrhenian sector of Basilicata, Italy. Among the taxa listed 44 are used as food or flavouring and 22 for animal alimentation; 7 taxa are involved in rituals especially connected with agriculture and plant growth. Click here for file
