# Supplementary material for: Food, flavouring and feed plant traditions in the Tyrrhenian sector of Basilicata, Italy
Source: J Ethnobiol Ethnomed. 2006 Sep 7;2:37. doi: 10.1186/1746-4269-2-37 (PMC1592457; doi:10.1186/1746-4269-2-37)
Supplement: Additional File 1 — Food, flavouring and feed folk uses of plants in the Tyrrhenian sector of Basilicata, Italy. Among the taxa listed 44 are used as food or flavouring and 22 for animal alimentation; 7 taxa are involved in rituals especially connected with agriculture and plant growth. [file 1746-4269-2-37-S1.pdf]

Table 1. Food, flavouring and feed folk uses of plants in the Tyrrhenian sector of Basilicata, Italy

Scientific name (voucher specimen)	*Local names*	*Parts used*	*Preparation*	*Popular uses*	*Citations N°*	*%*	*Fre-quen-cy*	*Current use*	*Users*	*Gathe-ring*	*Habi-tat*
Pteridophyta Equisetaceae											
Equisetum telmateja Ehrh. M1	Coda cavallina	Bu	Boiled buds	As food	1	0,3	R	No	F,O	Sp	Da
Angiospermae Dicotyledones											
Salicaceae											
Salix alba L.* M2	Salecòne	Br	Fresh branches	Fodder to strengthen young rabbits (2-3 months old)	2	0,59	R	Yes	P,F,O	Al	Ri
Juglandaceae											
Juglans regia L.° M3	Noce	Hu	Macerated in alcool	Flavouring to make the liqueur 'nocino'	4	1,19	C	Yes	P,F,O	Su	Cu
Fagaceae											
Quercus ilex L.* M4	Ìlece, elce	Fru	Fresh or dried fruits	Fodder for pigs	7	2,08	C	Yes	P,F,O	Fa	Tewo
Quercus virgiliana (Ten.) Ten.° M5, *Quercus pubescens* Willd.° M6	Cerza, cersa<br>Cerza, cersa	Fru	Roasted fruits	To make a substitute of coffee	1	0,3	R	No	F,O	Fa	Tewo
		Fru	Fresh or dried fruits	Fodder for pigs	7	2,08	Vc	Yes	P,F,O	Fa	
Moraceae											
Ficus carica L.° M7	Ficàra	Fru	Fresh fruits boiled and strained to obtain a food similar to honey	As food	2	0,59	R	Yes	F	Su	Cu
		Fru	Dried figs kept in wooden cases with *L. nobilis* leaves	As food	3	0,89	C	No	F,O	Fa, Wi	

Urticaceae											
Urtica dioica Mert. et Koch° M8	Ardica	Le, to	Crushed or boiled, mixed with bran	Fodder for chickens and turkeys	4	1,19	C	Yes	F,O	Al	Unc, Ru
		To	In omelettes	As food	3	0,89	C	Yes	F	Al	
Chenopodiaceae											
Beta vulgaris subsp. *vulgaris* L. M9	Bieta	Le	Cooked as a vegetable in "misca"	As food	4	1,19	Vc	Yes	P,F,O	Sp	Cu
Portulacaceae											
Portulaca oleracea L. M10	Purchiacca, vrìtti	To	In mixed salads	As food	5	1,48	C	Yes	F,O	Al	Kigd
Ranunculaceae											
Clematis vitalba L. M11	Turtagne, vitacchia	Bu	Boiled /fried lightly in olive oil or cooked in omelettes	As food	15	4,46	C	Yes	P,F,O	Sp	He, Ma
Lauraceae											
Laurus nobilis L. M12	Lauro	Le	In dry figs, fried in oil, with meat or to wrap 'fegatelli' in gelatine / with anchovies	Flavouring	6	1,78	Vc	Yes	P,F	Al	Cu; Da
Papaveraceae											
Papaver rhoeas L. subsp.	Papavero,	Le	In soups	Food to provoke sleep	1	0,3	R	No	F	Sp	Fi
rhoeas° M13	paparina	Le	As a cooked vegetable	As food in "misca"	1	0,3	Vc	Yes	P,F	Sp	
		Le	Mixed with bran	As fodder for chickens and rabbits	2	0,59	R	Yes	F	Sp	
		Fl	For floral "carpets" or to throw on the	Ritual use (for the Corpus Domini procession)	4	1,19	Vc	Yes	F,O	Sp	

			procession									
Cruciferae												
Nasturtium officinale R.	Crisciumuli,	To	In salad	As food	6	1,78	C	Yes	P,F,O	Sp,Su	Da
Br.° M14	crisciune	Ae p	Fresh or dry aerial parts	Fodder for pigs	2	0,59	C	Yes	P,F,O	Sp,Su	
Rosaceae											
Crataegus monogyna Jacq.° M15	Spina cerasola spina di piruna	Fru	Fresh or dry fruits	Fodder for pigs	3	0,89	R	No	P,F,O	Fall	Ma, Wo
Fragaria vesca L.° M16	Fragola	Fru	Fresh fruits	As food	4	1,19	C	Yes	P,F,O	Sp	Cle
Mespilus germanica L. M17	Nespolo	Fru	Fresh fruits	As food	1	0,3	R	Yes	P,F,O	Wi	Cu
Pyrus amygdaliformis Vill.° M18	Pirànio	Fru	Fresh or dry fruits	Fodder for pigs	1	0,3	R	No	P,F,O	Wi	Ma
Rubus ulmifolius Schott° M19	Spina, cèusa spina	Fru	Fresh fruits	As food	3	0,89	Vc	Yes	P,F,O	Su	Scr, Cle (Wo)
Sorbus domestica L. M20	Survi, sùgoro	Fru	Fresh or dried fruits	As food	2	0,59	R	No	P,F,O	Wi	Wo
Leguminosae											
Ceratonia siliqua L.°	Carrubo	Se	Dried seeds	As food	5	1,48	C	Yes	P,F,O	Al	Mema,
M21		Fru	Fresh or dried fruits	Fodder for livestock	17	5,06	C	Yes	F,O	Su	Co
Cicer arietinum L. M22	Cece	Se	Roasted	To make a substitute of coffee	2	0,59	C	No	F,O	Al	Cu
Pisum sativum L. subsp. *elatius* (Bieb.) Asch. et Gr.* M23	Oleca	Se	Tender seeds in soups	Food use	2	0,59	R	No	O	Su	Unc

Psoralea bituminosa L.* M24	Occhio di vojo	Ae p	Fresh plant or put in the hay	Fodder for cows	4	1,19	Vc	Yes	P,F,O	Sp, su	Rock, Suunc
Spartium junceum L.*	Spartu	St	Fresh stems	Fodder for rabbits	4	1,19	Vc	Yes	P,F,O	Al	Unc,
M25		Fl	Flowers thrown on the procession; floral carpets	Ritual use (for the Corpus Domini procession)	7	2,08	C	Yes	F,O	Sp, su	Shr
Rhamnaceae											
Ziziphus jujuba Mill.M26	Jujulu	Fru	Fresh fruits	As food	2	0,59	R	Yes	P,F,O	Su	Cu
Myrtaceae											
Myrtus communis L.° M27	Murtìdda, mortedda	Br (wo)	As skewers for preserving and pressing figs	Flavouring	3	0,89	C	Yes	P,F,O	Fa	Mema
Umbelliferae											
Crithmum maritimum L.* M28	Crìtini	To	In salad	As food	4	1,19	C	No	P,F,O	Al	Sero
Foeniculum vulgare		Le	As a cooked vegetable	As food in "misca"	7	2,08	Vc	Yes	P,F,O	Al	Esc,
Miller subsp. *piperitum* (Ucria) Coutinho° M29	Finocchio selvatico,	Fru	In olives, sausages with pepper/chili, tomato sauce	Flavouring	12	3,57	Vc	Yes	P,F,O	Su	Unc
	finucchiello, finocchietto finucchio,	Le	With sauge leaves, in pasta with sardines, in soups, in "ciambotta" and with rabbit	Flavouring	5	1,48	Vc	Yes	F,O	Sp	
		Ae p	Fresh aerial part	Fodder for rabbits	2	0,59	C	Yes	O	Sp	
Ericaceae											
Arbutus unedo L. M30	Sorve nataline, sorve selvagge survu pelusu	Fru	Fresh fruits	As food	3	0,89	R	Yes	P,F,O	Wi	Mema

Oleaceae											
Fraxinus ornus L.° M31	Milègro, Melègro	Br, le	Fresh leaves / branches	Fodder for goats	5	1,48	C	Yes	F,O	Sp,su,fa	Wo
Olea europaea L.° M32	Palma benedetta		A small branch is placed in the fields	Magic-religious use (to remove storms)	4	1,19	Vc	Yes	P,F,O	Sp	Cu
Boraginaceae											
Borago officinalis L. M33	Vurrania	Le, to	In fritters, in "misca", in soups, with "sanguinaccio"	As food	20	5,95	Vc	Yes	P,F,O	Sp,Wi	Unc, Cu
		Ae p	Fresh aerial parts	Fodder for pigs	3	0,89	C	Y	P,F,O	Sp	
Labiatae											
Mentha x *piperita* L. M34	Menta	Le	Fresh/dry (e.g. fried fish with boiled vinegar /garlic)	Flavouring	4	1,19	C	Yes	F,O	Al	Da
Origanum heracleoticum L.° M35	Rìgono, arìgano	Fl t	Dry minced flowery tops	Flavouring	6	1,78	Vc	Yes	P,F,O	Su	Moga
Satureja montana L. M36	Sarapuddu	Ae p	Dry minced flowery tops	Flavouring for boiled meats (goat)	1	0,3	R	Yes	F,O	Al	Moga
Teucrium polium L. subsp. *capitatum* (L.) Arcang.* M37		Ae p	Fresh/dry minced flowery tops	Flavouring	1	0,3	R	No	O	Al	Drpa, Ga
Plantaginaceae											
Plantago major L.°M38	Centonerve	Se	Fresh/dry seeds	Fodder for goldfinches	1	0,3	C	No	O	Su	Unc,Me
Compositae											
Cichorium intybus L. M39	Cicoria	Le	As cooked vegetable in "misca"	As food	11	3,27	Vc	Yes	P,F,O	Al	Unc, Cu

Helichrysum italicum (Roth) G.Don fil.* M40		Ae p	Fresh/dry minced aerial parts	Flavouring for sauces	1	0,3	R	No	F,O	Su	Ga
Inula crithmoides L.* M41	Crìtini	To	In salad	As food	4	1,19	C	No	F,O	Al	Sero
Picris echioides L. M42 *P. hieracioides* L. M43	Purcinara Purcinara	Le	Cooked in "misca", frequently in wartime	As food	3	0,89	Vc	Yes	P,F,O	Sp	Cu, Unc
		Ae p	Fresh aerial parts	Galactagogue fodder for rabbits	1	0,3	R	Yes	P,F,O	Al	
		Ae p	Fresh aerial parts	Fodder for pigs	2	0,59	C	Yes	P,F,O	Al	
Reichardia picroides (L.)	Galazzina,	Le	As cooked vegetable	As food in "misca"	3	0,89	C	Yes	P,F,O	Sp	Uncdr,
Roth° M44	grattalingua,	Le	In soups	As food	2	0,59	Vc	Yes	P	Sp	Wa
	lattucetta	Ae p	Fresh aerial parts	Fodder for rabbits	2	0,59	C	Yes	F,O	Al	
Sonchus sp.pl. (*S. arvensis* L.° M45, *S. asper* (L.) Hill° M46, *S. oleraceus* L.* M47, *S. tenerrimus* L.* M48)	Juncia, junci	Le	As cooked vegetable in "misca" and in soups	As food	9	2,67	Vc	Yes	P,F,O	Al	Unc, Cu; Wa (*S. tener-rimus*)
		Le	Salads	As food	2	0,59	R	Yes	F	Sp	
Tussilago farfara L. M49	Ugna	Ae p	Minced aerial part	Fodder for pigs (with bran)	4	1,19	C	No	P,F,O	Sp-Fa	Dacl
Urospermum picroides (L.) Scop. ex F.W. Schmidt M50	Erba piatta	Le	As cooked vegetable in "misca" and in soups	As food	1	0,3	R	Yes	P	Wi,Sp	Unc, Cu

Angiospermae Monocotyledones

Liliaceae

Asparagus acutifolius L. M51	Spàragi	Tur	In omelettes, boiled, in "pasta"	As food	10	2,97	Vc	Yes	P,F,O	Sp	Mema, Tewo
Asphodelus microcarpus Salzm. et Viv.* M52	Sciòddule;	Tub	Fresh/dry tubers	Fodder for pigs	1	0,3	C	No	F,O	Al	Ga, Pa
	catània (the	Le	Fresh/dry leaves	Fodder for cows and goats	3	0,89	C	No	F,O	Al	
	flowers)	Ae p	The abundant flowering is interpreted as good future harvests in agricolture	Ritual use (to make omens)	1	0,3	C	Yes	P,F,O	Sp	
Asphodeline liburnica (Scop.)Rchb.* M53	Sbaràvule	Bu	In omelettes and in batter (fritters)	As food	5	1,48	R	No	P,F,O	Sp	Drro
Leopoldia comosa (L.) Parl.° M54	Cipuddini	Bul	In omelettes; fried with bacon, potatoes, pig's fat	As food (bulbs flavoured with garlic, chilli pepper, mint, oregano)	10	2,97	C	Yes	P,F,O	Sp	Uncdr
		Bul	Boiled, baked with lamb	As food (for the seasoning see above)	8	2,37	R	Yes	P,F,O	Sp	
		Le	In omelettes	As food	1	0,3	Vc	Yes	P,F	Sp	

Graminaceae

Cynodon dactylon (L.)	Gramigna	Rh	Crushed to extract flour	In times of famine added to wheat flour	1	0,3	C	No	F,O	Al	Unc,
Pers.* M55		Rh	Boiled	As food in times of famine	1	0,3	C	No	F,O	Al	Cu, Ro
Digitaria sanguinalis (L.) Scop.* M56	Pulicastrieddu, p. femmenino	Ae p	Fresh aerial parts	Fodder for rabbits	5	1,48	C	Yes	P,F,O	Sp	Unc, Cu
Hordeum vulgare L. M57	Orzo	Fru	Roasted	To make a substitute of coffee	9	2,67	Vc	No	F,O	Al	Cu

Lolium multiflorum Lam. subsp. *gaudini* (Parl.) Sch. et Th.* M58, *L. perenne* L.* M59	Gioglio, sciuoglio Gioglio, sciuoglio	Fru, fl, le	The small ears were counted, reciting: "Much, little, nothing"and the last small ear indicated whether a love was reciprocated	Ritual use (for make omens)	8	2,37	Vc	No	F,O	Sp,Su, Fa	Unc, Cu
Setaria sp.* M60	Pulicastrieddu masculino	Ae p	Fresh aerial part	Fodder for cows	1	0,3	R	No	P,F	Sp,su	Unc, Cu
Araceae											
Arum italicum Miller* M61	Pizzicuorno, vrai	Le	Cooked leaves	Fodder for pigs	1	0,3	R	Yes	P	Al	Ma, He, Cu
		Rh	Fresh/dry rhizomes	Fodder for pigs	5	1,48	R	Yes	P,F,O	Al	
		Ae p, rh	Abundant flowers and fruits are an omen of good future in agriculture	Ritual use (for omens)	3	0,89	R	Yes	P,F,O	Fa-Wi	

Shortenings (table I): * = plant not cited in Caneva et al., 1997; Pieroni et al., 2002a; 2002 b; ° = plant cited in the above mentioned papers but at least one use new or different in comparison with them **Citations** The % is referred to a total number of citations (336) **Parts used** Ae p = aerial part; br = branches; bu = buds; bul = bulb; fl = flowers; fl t = flowery tops; fru = fruits; Hu = husk; rh = rhizomes; se = seeds; st = stems; to = tops; tub = tubers; tur = turiones; wo = wood **Use** C= common; Vc= very common; R= rare **Users** P= personal; F= familiar; O= mentioned by others **Period of gathering** Sp= spring; Su= summer; Fa= fall; Wi= winter; Al=always **Habitat** Cle **=** Clearings; Co = Coasts; Cu= cultivated areas; Da = damp areas; Dacl = damp clayey areas; Drpa = dry pastures; Drro = dry rocksy slopes; Esc = escarpments; Fi = fields; Ga = garrigues; Kigd = kitchen gardens; He = hedges; Ma = maquises; Me = meadows; Mema = Mediterranean maquis; Mewo = mesophilous woods; Moga = mountain garrigues; Pa = pastures; Ri = along rivers; Ro = roadsides; Rock = rocky slopes; Ru = ruins; Scr = scrubland; Sero = sea rocks; Sh = shrubby areas; Suunc = sunny uncultivated areas; Tewo = termophilous woods; Unc = uncultivated areas; Uncdr = uncultivated dry areas; Wa = walls; Wo = woods
